# Pediatric Clinical Images Without Consent: A Governance Gap in the Long-Term Reuse of Health Data in Digital Health Ecosystems

**DOI:** 10.2196/94952

**Published:** 2026-06-01

**Authors:** Iwona Bujek

**Affiliations:** 1 Poznan University of Medical Sciences Poznan Poland; 2 GGZ Voor Elkaar, Mental Health Institution Utrecht The Netherlands

**Keywords:** pediatric clinical images, informed consent, digital health governance, legacy data, medical education, artificial intelligence, patient privacy, data ethics

## Abstract

Digital health governance frameworks have primarily focused on prospective safeguards, including informed consent at the point of data collection, lawful processing, and data security. Comparatively less attention has been devoted to the long-term circulation of legacy clinical materials, particularly pediatric clinical images reused across educational and digital infrastructures. This viewpoint examines governance challenges associated with the prolonged educational and digital reuse of pediatric clinical images without identifiable evidence of consent. Drawing on a longitudinal case spanning more than 3 decades (1991-2026), this article illustrates how clinical images may continue circulating across textbooks, educational repositories, conference materials, e-books, and online teaching platforms long after their original creation and publication context. The case is informed by archival educational materials, institutional correspondence, publisher communications, and formal regulatory findings, including a decision issued by the Polish Patient Rights Ombudsman confirming continuing violations related to dissemination of intimate pediatric clinical images without identifiable consent. This article argues that current digital health governance frameworks remain insufficiently equipped to address persistence, traceability, provenance, and coordinated withdrawal of legacy clinical materials once they enter distributed educational ecosystems. Fragmented accountability across health care institutions, publishers, educational systems, libraries, repositories, and digital platforms may allow sensitive clinical materials to remain accessible despite regulatory intervention or removal requests. The article further discusses how publicly accessible educational materials may become incorporated into downstream artificial intelligence and machine learning ecosystems through digitization, aggregation, web scraping, and secondary dataset reuse. In this context, unresolved historical consent deficiencies may become embedded within artificial intelligence–enabled infrastructures without effective provenance tracking or remediation mechanisms. To address these limitations, this viewpoint proposes a lifecycle-oriented governance framework emphasizing long-term consent traceability, provenance-aware dissemination systems, verification checkpoints before reuse or republication, periodic review of legacy educational archives, and coordinated cross-platform withdrawal procedures.

## Introduction

Clinical images are integral to medical education, research, and clinical communication. In digital environments, however, their circulation may extend far beyond the original context of care or publication. Once incorporated into textbooks, conference materials, online repositories, educational platforms, and digital archives, clinical images may persist for decades and move across jurisdictions, institutions, and technological systems [1-6]. Digital health governance frameworks increasingly address the ethical management of health data, secondary data use, and artificial intelligence (AI) applications in medicine [7-11]. These frameworks primarily emphasize prospective safeguards, including informed consent, lawful processing, data security, and accountability [12-18]. By contrast, comparatively less attention has been devoted to the long-term persistence and downstream reuse of legacy educational materials after their initial publication. This governance gap is particularly significant for clinical images because identifiability may persist even after partial anonymization. Existing publication guidance emphasizes that masking the eye region alone may not adequately protect anonymity and that consent should be obtained whenever identifiability cannot reasonably be excluded [1-6]. Recent studies similarly report uncertainty among clinicians, publishers, and editors regarding acceptable standards for publication and reuse of patient photographs in digital environments [3,4]. Within educational ecosystems, responsibility for consent verification is frequently fragmented. Authors, publishers, universities, libraries, repositories, and digital platforms operate within partially overlapping but insufficiently coordinated mandates. Publishers may rely primarily on author declarations without independent validation, while educational institutions may not systematically evaluate whether consent remains valid across subsequent editions, formats, or digital dissemination channels [1,2,4]. Pediatric clinical images require particular ethical attention because they originate in contexts characterized by vulnerability, dependency, and limited autonomy [19-22]. Individuals depicted in such materials may later encounter their own images in educational or online settings decades after image creation. These situations raise questions regarding privacy, dignity, autonomy, and long-term control over personal data. This viewpoint uses a longitudinal case involving pediatric clinical images disseminated without identifiable consent records as an analytical reference point for examining governance limitations related to persistence, reuse, accountability, provenance, and withdrawal of legacy clinical materials within contemporary digital health ecosystems. Rather than aiming to establish prevalence, the article uses this illustrative case to explore broader governance challenges associated with the long-term circulation of sensitive clinical materials across educational and digital infrastructures.

## A Documented Longitudinal Case of Educational Reuse

The governance concerns discussed in this article are illustrated through a longitudinal case spanning more than 3 decades (1991-2026). The discussion draws on educational materials, institutional correspondence, publisher communications, and regulatory findings related to the dissemination of pediatric clinical images across educational and digital infrastructures. The case came to attention in 2023 when an adult medical professional recognized his own pediatric clinical images in publicly accessible educational materials. The images originated from hospital-based clinical care during childhood and adolescence in Poland and had subsequently circulated through academic textbooks, conference presentations, lecture materials, e-books, digital repositories, and online educational platforms. The identified materials included more than 40 editions and reprints of academic medical textbooks in addition to conference resources, downloadable slide collections, and publicly accessible online teaching materials. Dissemination occurred across both print and digital environments, including institutional libraries, educational syllabi, online repositories, and multimedia-sharing platforms. The images included full-body representations of children approximately 3 to 17 years of age. In several instances, full facial features and intimate anatomical regions remained visible. Some materials contained no anonymization, while others used partial masking techniques such as covering the eye region. However, these measures did not reliably prevent recognition where additional facial, bodily, or contextual characteristics remained visible [1,3,5,6]. No identifiable evidence of informed consent for publication or long-term educational reuse could be located across the analyzed materials. The affected individual reported no prior awareness of the existence or dissemination of these materials before 2023. The case is informed by archival materials, institutional correspondence, and regulatory findings, including a decision issued by the Polish Patient Rights Ombudsman on August 27, 2025, concerning continued dissemination of intimate pediatric clinical images without verifiable consent. Identifying institutional and personal details remain intentionally limited because of ongoing legal and privacy considerations. Despite regulatory intervention, the materials remained accessible in 2026 across university libraries, academic reading lists, digital repositories, and previously distributed print editions. Institutional responses were fragmented and inconsistent, and no coordinated mechanism existed to support comprehensive provenance tracking, retrospective consent verification, or coordinated withdrawal across platforms and institutions.

### Fragmented Accountability Across Educational Ecosystems

This case illustrates how sensitive clinical materials may persist across educational and digital infrastructures for decades without effective mechanisms for consent traceability, provenance verification, or coordinated remediation. The continued circulation of the identified images reflected fragmentation of responsibility across health care institutions, publishers, educational systems, libraries, repositories, and digital dissemination platforms. No single actor retained both the authority and operational capacity to ensure comprehensive withdrawal once the materials entered distributed educational circulation. The case also highlights the temporal dimension of harm associated with pediatric clinical data. The affected individual became aware of the dissemination only decades later through self-recognition in publicly accessible educational materials. Importantly, identification and subsequent investigation depended heavily on the individual’s professional expertise and digital literacy. Without the ability to recognize the clinical context and trace dissemination pathways, the unauthorized reuse might never have been identified. These observations suggest that detection of historical consent deficiencies may depend less on institutional safeguards than on the ability of affected individuals to recognize and investigate unauthorized reuse. They also illustrate how ethical and regulatory recognition of violations does not necessarily translate into effective remediation once materials become embedded within decentralized educational ecosystems. Historical differences in professional norms, publication practices, archival governance, and documentation standards should also be acknowledged [1,2]. Consent procedures and institutional governance frameworks have varied substantially across jurisdictions and historical periods. Nevertheless, such variability does not eliminate the relevance of contemporary governance responsibilities when legacy materials remain publicly accessible and continue circulating within modern digital and educational environments.

#### AI-Enabled Secondary Reuse and Provenance Challenges

These governance limitations take on additional significance within contemporary AI ecosystems. Educational clinical materials may enter machine learning pipelines through web scraping of publicly accessible repositories, digitization of legacy textbooks, downstream aggregation, secondary dataset reuse, and incorporation into multimodal training datasets [7-11,23,24]. Publicly accessible educational repositories may therefore become indirect sources of sensitive clinical materials within AI development infrastructures. Where consent provenance remains unclear or undocumented, historical ethical deficiencies may become embedded within AI-enabled systems without effective visibility, traceability, or remediation mechanisms. Current governance initiatives, including the European Health Data Space, the European Union AI Act, and emerging international AI governance frameworks, remain primarily oriented toward prospective data collection and processing [10,11]. Comparatively less attention has been devoted to legacy educational materials already circulating across distributed institutional and technological infrastructures. The governance issues illustrated by this case therefore extend beyond traditional publication ethics. They raise broader questions concerning downstream reuse, provenance-aware data governance, institutional accountability, and operational mechanisms for retrospective remediation within evolving AI-enabled health data ecosystems.

#### Toward Lifecycle Governance Frameworks

The governance limitations illustrated by this case suggest the need for lifecycle-oriented approaches capable of addressing persistence, reuse, provenance, reidentification risk, and withdrawal across evolving technological and institutional environments.

Such approaches may include (1) long-term retention of verifiable publication consent documentation; (2) separation of publication consent from treatment consent; (3) verification checkpoints before republication or digital redistribution; (4) provenance-aware dissemination and metadata systems; (5) periodic institutional review of legacy educational archives; and (6) coordinated cross-platform withdrawal procedures involving health care institutions, publishers, universities, libraries, repositories, and digital platforms.

Table 1 summarizes a proposed lifecycle governance framework for clinical images based on governance limitations illustrated through the documented case.

The framework emphasizes that governance responsibilities should not end at the point of initial publication. Instead, accountability mechanisms may need to extend across the full lifecycle of educational clinical materials, including subsequent redistribution, digitization, archival persistence, AI-enabled downstream reuse, and remediation processes.

**Table 1:** Lifecycle governance framework for clinical images.

Lifecycle stage	Primary responsible actors	Observed governance failures	Proposed governance mechanisms	Accountability/verification mechanism
Creation	Clinicians, hospitals, health care institutions	Absence, loss, or nonretention of documented publication consent; conflation of treatment and publication consent	Separate publication consent from treatment consent; require durable retention of consent documentation; document scope and duration of reuse permissions	Health care institutions maintain auditable consent records subject to regulatory review
Initial publication and dissemination	Authors, publishers, journal editors, educators	Reliance on author declarations without independent consent verification; inadequate review of identifiability risks	Require verifiable consent documentation before publication and republication; implement standardized consent review procedures for clinical images	Publishers and editors verify and retain consent documentation before dissemination
Secondary educational reuse	Universities, libraries, educational repositories, professional societies	Reuse across editions, courses, repositories, and presentations without renewed consent verification or provenance review	Introduce verification checkpoints before republication, digitization, or educational redistribution; require provenance-aware metadata systems	Educational institutions and repositories conduct periodic compliance and provenance review
Digital persistence and redistribution	Digital repositories, e-book platforms, search engines, online educational platforms	Continued circulation after original publication; fragmented dissemination across digital infrastructures; absence of coordinated oversight	Implement consent-linked metadata, provenance tracking, and interoperable removal protocols across platforms	Platform operators maintain traceable provenance and respond to validated removal requests
Withdrawal and remediation	Regulators, publishers, universities, repositories, platform operators	Incomplete withdrawal despite formal takedown orders; inconsistent institutional responses; persistence of distributed copies	Establish coordinated cross-platform withdrawal procedures and remediation protocols spanning print and digital systems	Regulatory authorities coordinate compliance monitoring and institutional remediation
Cross-cutting governance	All institutional actors	Fragmented accountability; unclear distribution of responsibility across systems and jurisdictions	Develop lifecycle-based governance frameworks with clearly defined institutional responsibilities, auditability, and oversight mechanisms	Shared governance agreements and periodic external review across participating institutions

## Conclusions

This viewpoint illustrates how pediatric clinical images may persist across educational and digital infrastructures for decades without identifiable consent records, even after formal regulatory intervention. The documented case highlights governance limitations emerging not only at the point of initial publication, but also through the long-term absence of mechanisms capable of supporting consent traceability, provenance verification, coordinated withdrawal, and accountability across distributed systems. Current digital health governance frameworks remain primarily oriented toward prospective data collection and processing. However, the continued circulation of legacy clinical materials suggests the need for lifecycle-based governance approaches capable of addressing persistence, reuse, reidentification risk, and downstream dissemination within evolving educational and AI-enabled ecosystems [13,16-18,23-25]. This viewpoint proposes a lifecycle governance perspective for the long-term management of sensitive clinical materials, emphasizing retrospective consent verification, provenance-aware dissemination, reuse validation, and coordinated remediation mechanisms spanning health care institutions, publishers, educational systems, repositories, and digital platforms.

## Abbreviations

AI: artificial intelligence
